# Multiple Therapeutic Modalities in Intermediate-High-Risk Pulmonary Embolism: A Case Report

**DOI:** 10.7759/cureus.77543

**Published:** 2025-01-16

**Authors:** Sandra OIiveira-Mendes, Rafaela Lopes Freitas, Joana Simões, Carolina Guedes

**Affiliations:** 1 Internal Medicine, Hospital Pedro Hispano, Matosinhos, PRT

**Keywords:** multidisciplinary discussion, percutaneous thrombectomy, pulmonary embolism, rescue therapy, thrombolysis

## Abstract

Pulmonary embolism (PE) is a significant cardiovascular condition requiring stratified management, particularly in intermediate-high-risk cases where hemodynamic instability is absent but markers of severity are present. This report presents the case of a 54-year-old female with malignancy-associated intermediate-high-risk PE complicated by severe hypoxemic respiratory failure and high bleeding risk. The patient underwent multiple advanced interventions, including percutaneous thrombectomy, catheter-directed thrombolysis, and reduced-dose systemic thrombolysis, due to persistent thrombotic burden and respiratory insufficiency. The case underscores the challenges of managing PE in the context of advanced malignancy and comorbidities, necessitating a balance between thrombosis resolution and minimizing hemorrhagic risk. The therapeutic approach was partially guided by the "MOPETT trial" findings, demonstrating the efficacy and safety of reduced-dose thrombolysis in moderate PE cases. Despite achieving significant improvements in respiratory function, the patient’s advanced-stage malignancy and declining functional status ultimately limited oncological treatment options, transitioning care to a palliative focus. This case emphasizes the critical role of individualized therapeutic strategies that address both the acute thrombotic event and the broader oncological context, aiming to optimize patient outcomes while considering quality of life and palliative care needs. It also highlights the importance of multidisciplinary collaboration in managing complex PE cases.

## Introduction

Venous thromboembolism, particularly pulmonary embolism (PE), is a common cardiovascular syndrome, and its management depends on appropriate patient stratification from the outset [1].

In patients with intermediate-high-risk PE, there is no hemodynamic instability "ad initium," but markers of severity are present, such as right ventricular dysfunction, assessed by echocardiography or computerized tomography pulmonary angiography (CTPA) for right ventricle strain and elevated cardiac biomarkers (troponin and/or NT-proBNP levels). In these cases, rigorous monitoring is essential due to the risk of decompensation and the potential need for rescue therapy [1,2].

Salvage thrombolysis is indicated in patients with severe respiratory insufficiency (RI) or clinical deterioration. This refers to systemic thrombolytic therapy in life-threatening PE cases as a last resort, typically when initial therapies such as anticoagulation have failed [3].

For those with a high bleeding risk, systemic thrombolysis generally should be avoided due to a significant risk of major bleeding, and percutaneous thrombectomy (PT) or reduced-dose thrombolysis may be considered [1,4,5]. PT offers a mechanical means to remove clots, reducing reliance on thrombolytic agents and minimizing bleeding complications [6].

We present the case of a patient with intermediate-high-risk PE, severe RI, and a high bleeding risk who underwent two rescue therapies.

## Case presentation

A 54-year-old female patient with an Eastern Cooperative Oncology Group (ECOG) performance status [7] of 0 and a history of obesity grade 3, smoking, and previous thrombotic events - deep vein thrombosis of the left lower limb and PE five years earlier with no cause established - was maintained on direct oral anticoagulant therapy with good compliance.

She was admitted with a one-week history of abdominal pain and distension. Investigations led to the diagnosis of an abdominal tumor mass with peritoneal carcinomatosis. Percutaneous biopsy of the tumor mass and histopathological examination identified mucinous adenocarcinoma, though the primary origin (gynecological or gastrointestinal) remained unclear. She was discharged with referrals to gynecology and scheduled for endoscopic studies on an outpatient basis.

**Figure 1:**
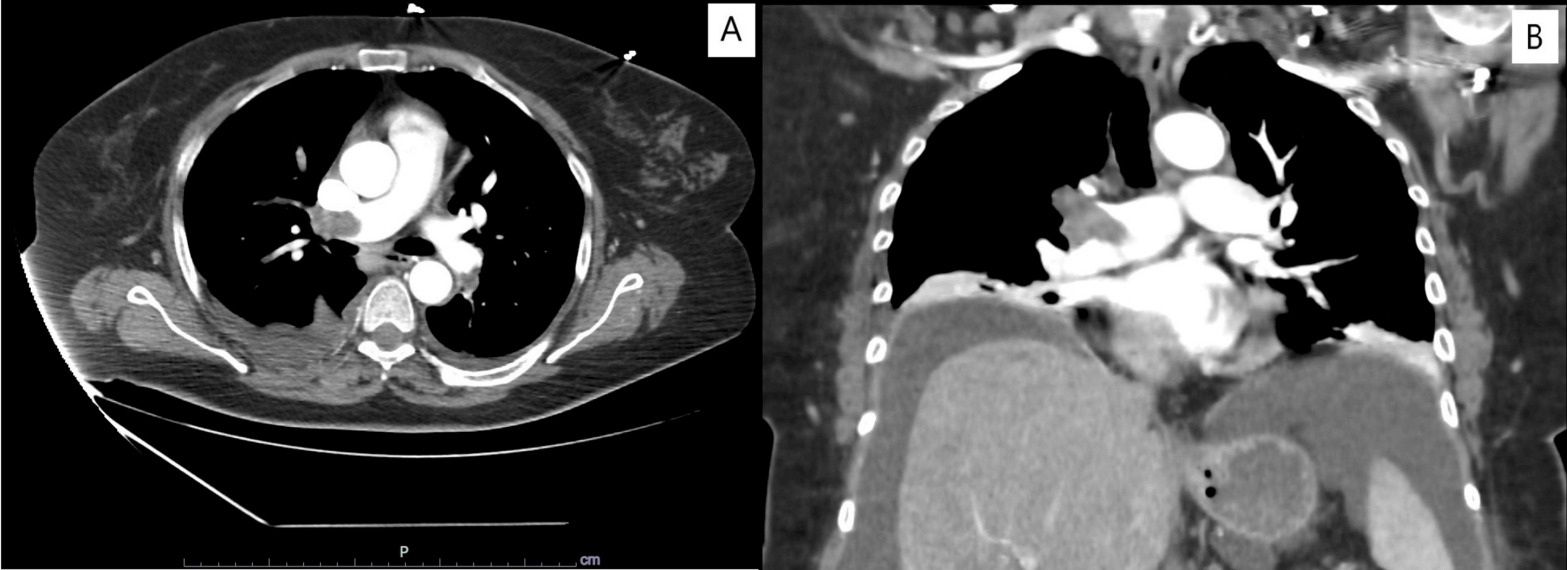
Initial thoracic CTPA showing bilateral PE (panel A – axial view, panel B – coronal view) CTPA: computed tomography pulmonary angiography, PE: pulmonary embolism

Approximately two weeks after discharge, she arrived at the emergency department with sudden-onset dyspnea, signs of respiratory distress, and severe hypoxemic respiratory failure. She was tachycardic but normotensive, and bedside cardiac ultrasonography showed right ventricular dilation. Given the high pre-test probability for PE, a thoracic CTPA confirmed bilateral thromboembolism with evidence of right heart strain (Figure 1).

She presented with microcytic/hypochromic anemia (hemoglobin 10.2 g/dL) and an undetermined malignancy. She was on anticoagulation therapy, which raised concerns about the possibility of active bleeding of the tumoral mass, gastrointestinal tract, or occult primary tumor. The anticoagulation strategy was switched to low molecular weight heparin to reduce bleeding risk and the worsening of the anemia. Hemoglobin remained stable. Non-invasive ventilation and high-flow nasal oxygen therapy were initiated for respiratory dysfunction management.

**Figure 2:**
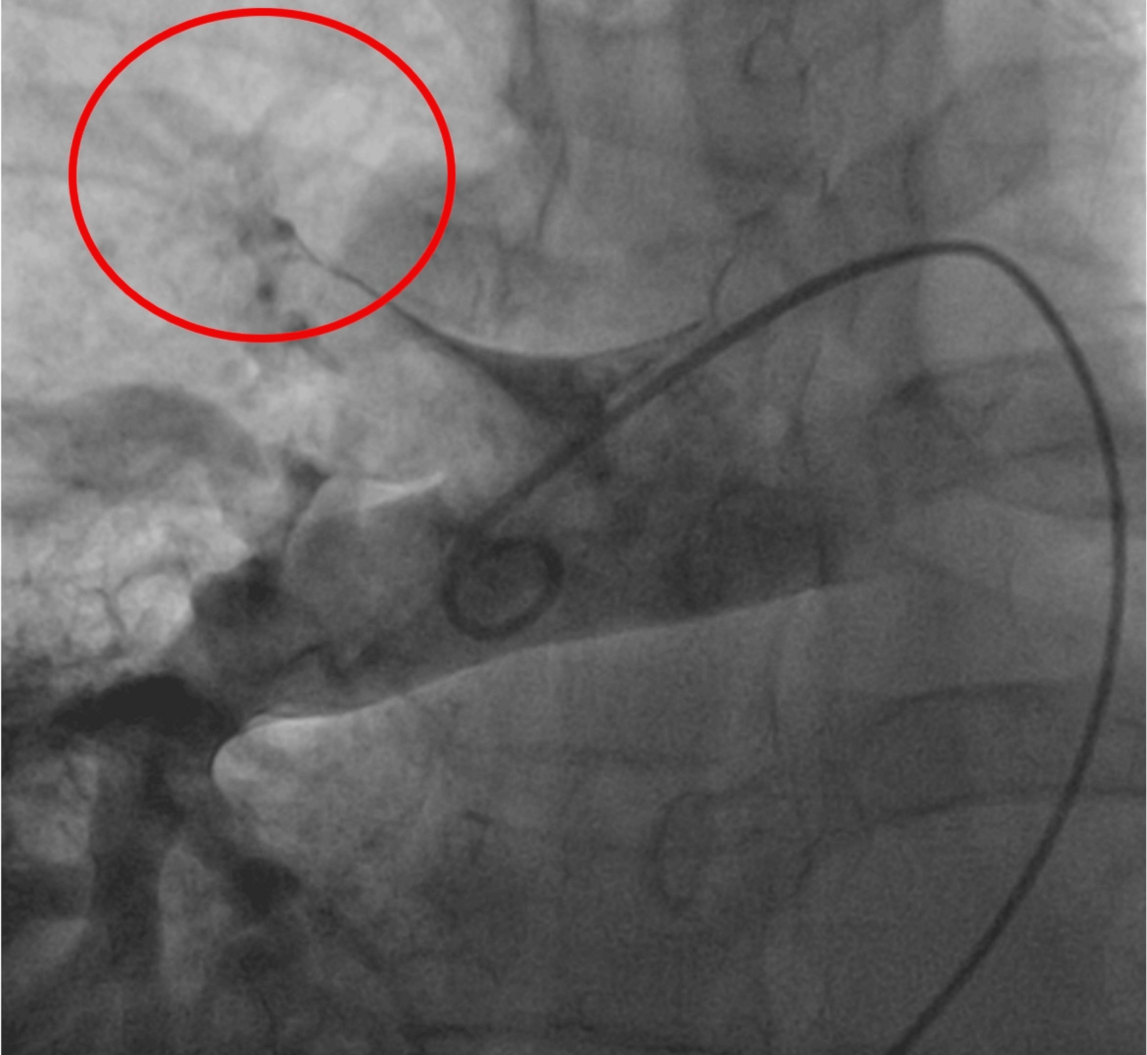
Selective pulmonary angiography showing occlusion of the right upper lobar branch (red circle)

Respiratory failure was challenging to manage, requiring continuous non-invasive ventilation. The ratio of partial pressure of oxygen in arterial blood (PaO2) to the fraction of inspiratory oxygen concentration (FiO2) - PaO2/FiO2 - was persistently around 100. Following consultation with a specialized center, the patient underwent PT. Angiography demonstrated a complete occlusion of the right upper lobar branch (Figure 2).

**Figure 3:**
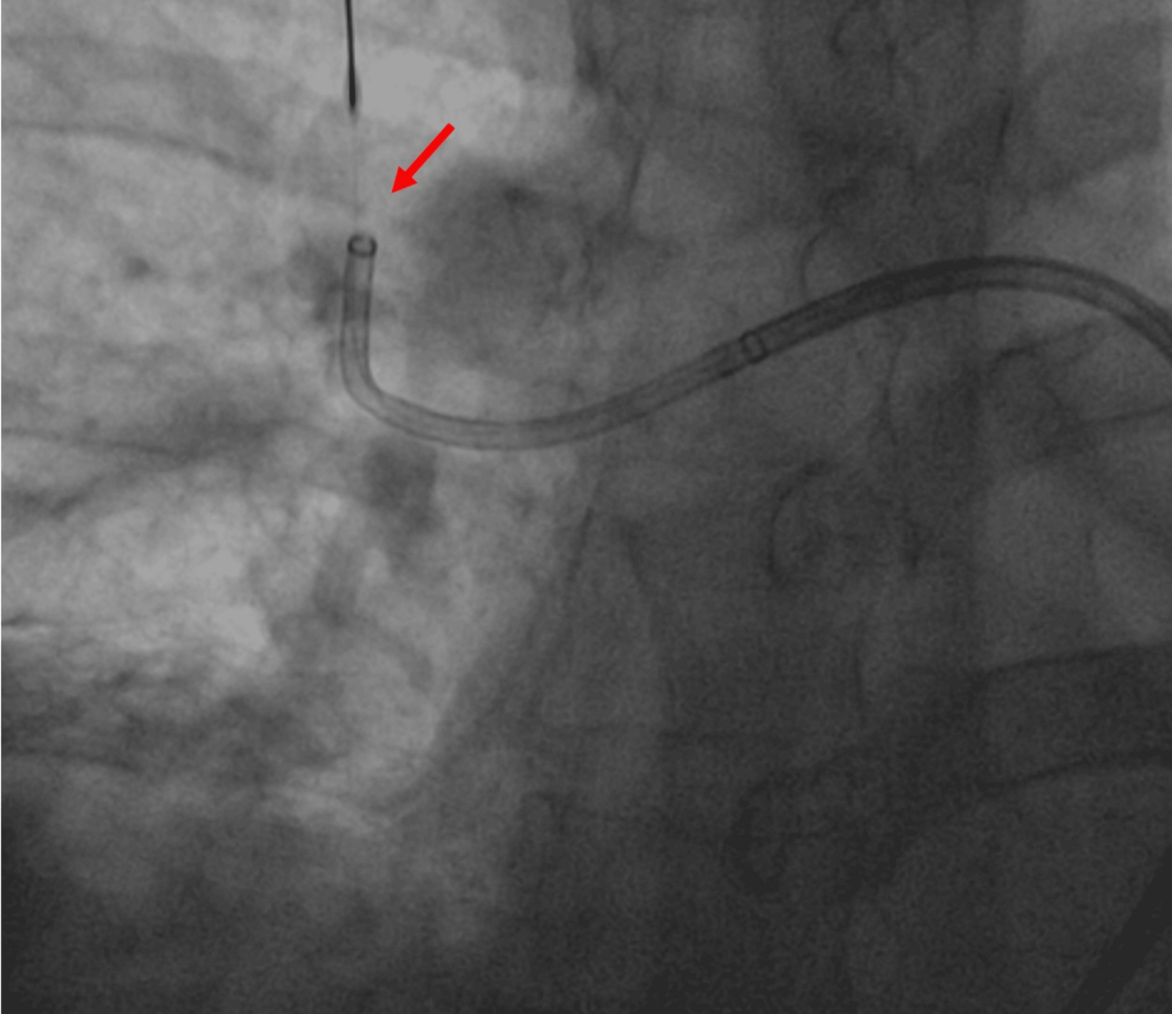
Pulmonary angiography: percutaneous thrombectomy with the assistance of a thrombus separator (red arrow)

**Figure 4:**
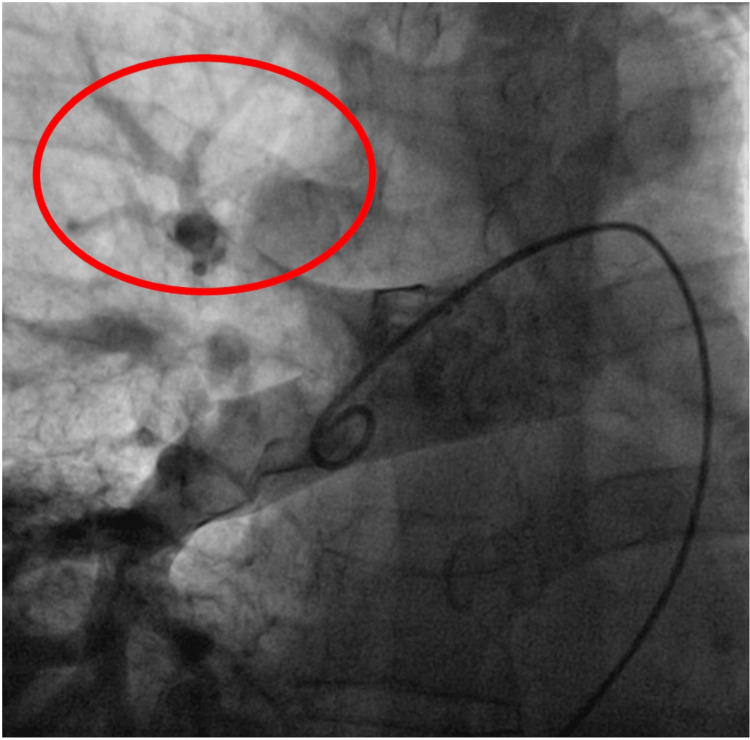
Pulmonary angiography: result showing reperfusion of the treated segment (albeit with residual thrombus)

Thrombus aspiration was performed (Figure 3), achieving partial revascularization (Figure 4). Local alteplase infusion was initiated during the procedure (a bolus of 3 milligrams (mg) followed by a continuous infusion at 1 mg/hour for six hours).

The thrombus persisted at the 72-hour imaging reassessment with CTPA, and there was no improvement in RI. Rescue thrombolysis was initiated following the protocol of the "MOPETT trial," administering a total of 50 mg of alteplase (maximum dose adjusted to body weight) through a 10 mg bolus followed by an infusion of 40 mg over a two-hour period [6].

Given the lack of significant clinical improvement, the patient underwent a second pulmonary angiography when conditions permitted. The procedure confirmed the persistence of a non-occlusive thrombus in the right upper lobar artery. The thrombus was successfully aspirated, resulting in a favorable final angiographic outcome.

**Figure 5:**
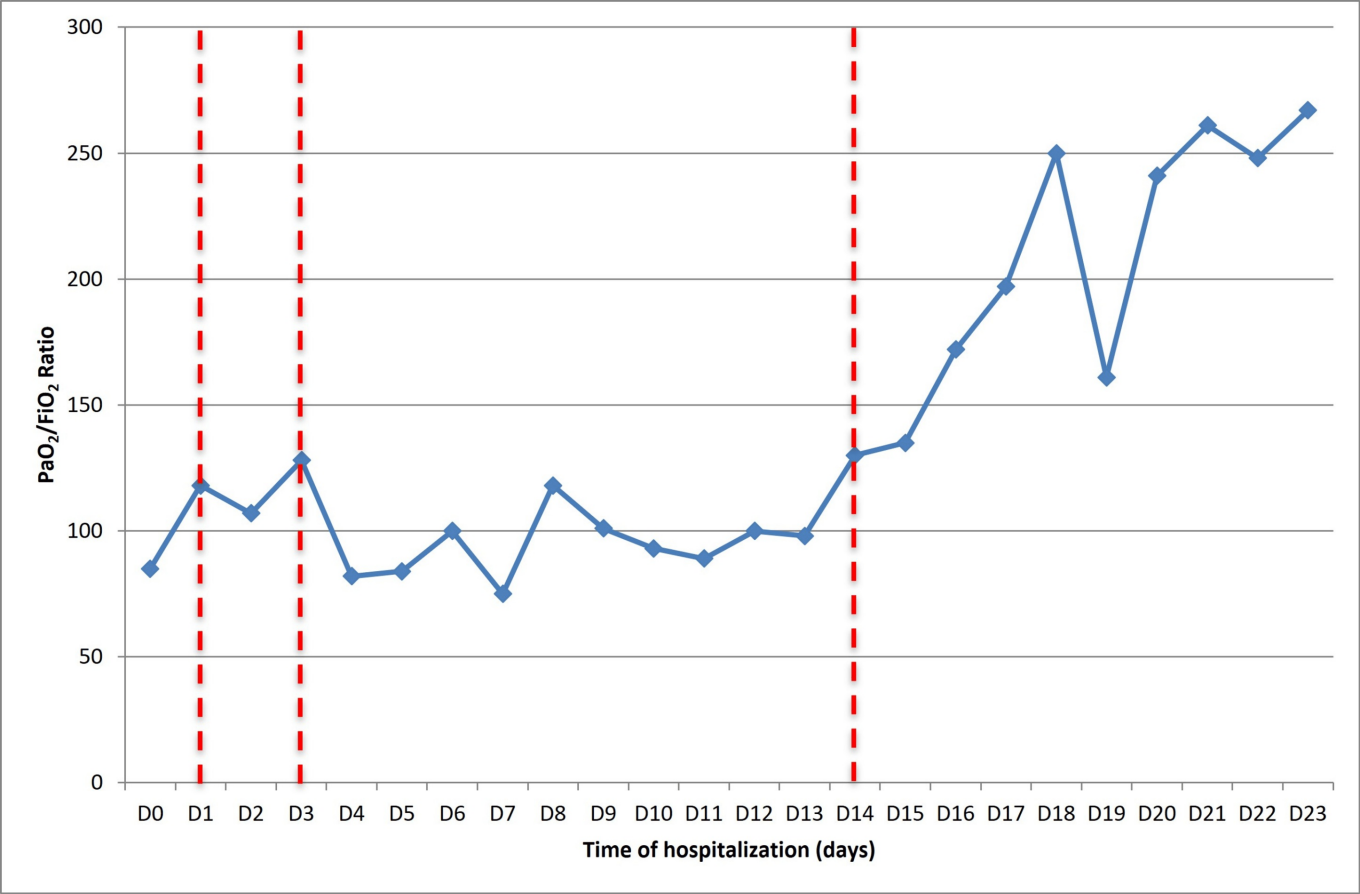
Evolution of respiratory dysfunction according to the calculation of the PaO2/FiO2 ratio throughout hospitalization. The vertical dashed red lines represent the therapies the patient underwent: the first thrombectomy and direct thrombolysis on day 1 of hospitalization, low-dose thrombolysis on the third day of hospitalization, and the second thrombectomy on the 14th day of hospitalization PaO2/FiO2: partial pressure of oxygen in arterial blood to the fraction of inspiratory oxygen concentration

A significant improvement in RI was observed following the second PT (Figure 5). This allowed for a reduction in oxygen therapy support to a conventional nasal cannula during daytime hours and non-invasive ventilation in CPAP mode during the nighttime, enabling the continuation of oncological disease investigation.

After managing multiple complications arising from the rapid growth of the tumor mass, the patient underwent the initially planned endoscopic evaluations. Without pathological findings, the neoplasm was presumed to originate from the ovary.

However, after assessment by a multidisciplinary oncology team, the patient was determined to be ineligible for systemic therapy due to the advanced stage of the disease and declining functional status. Management was subsequently transferred to the palliative care team.

## Discussion

Thrombolysis is a well-established therapeutic option for managing massive PE and symptomatic PE without hemodynamic instability. However, systemic thrombolysis is not recommended for patients with intermediate-high-risk PE due to the increased risk of bleeding complications, which often outweigh the potential hemodynamic benefits [1,2]. In such cases, catheter-directed reperfusion therapy should be considered, especially when systemic thrombolysis is contraindicated [8,9].

For patients with a particularly high thrombotic burden, mechanical thrombectomy alone may be insufficient to achieve significant clinical improvement. The lungs exhibit a unique sensitivity to thrombolytic agents, prompting investigations into the safety and efficacy of thrombolysis using reduced tissue plasminogen activator doses. The "MOPETT trial" demonstrated that "safe dose" thrombolysis is effective and safe for treating moderate PE by reducing pulmonary artery pressures and bleeding complications compared to full-dose thrombolysis. Also, it improved functional outcomes in patients with moderate PE. This approach was associated with an enhanced safety profile, reducing bleeding complications risk [5].

This case highlights the challenges of managing intermediate-high-risk PE in the context of malignancy and other comorbidities. The patient presented with severe hypoxemic respiratory failure due to bilateral pulmonary thromboembolism, which required advanced interventions, including percutaneous thrombectomy and thrombolysis. Despite initial treatments, persistent thrombus necessitated multiple salvage therapies. In selected cases, these therapies play a significant role in reducing thrombotic burden and improving clinical outcomes. The decision-making process was influenced by the patient’s high hemorrhagic risk, anemia, and malignancy of unknown etiology, underscoring the need for individualized approaches.

Multidisciplinary discussion is crucial in managing complex cases, and it must be comprised of experts such as pulmonary embolism response teams [10], in which internists, cardiologists, and interventional cardiologists can be included.

Our ultimate goal was to enhance the patient's respiratory function to facilitate progression through the diagnostic pathways. In our reality, there is no availability of treatment without a histopathological diagnosis that confirms the primary origin. Once the endoscopic evaluation excluded the gastrointestinal origin, it would be assumed that the tumor was of gynecological origin, and then the patient would be evaluated for systemic therapy. The progression of the tumor and the patient’s deteriorating functional status to an ECOG [7] of 3 further constrained therapeutic possibilities, ultimately precluding systemic cancer therapy.

## Conclusions

This case highlights the critical need to balance assertive management of PE with consideration of the patient’s overall oncological condition. Malignancy-associated thrombosis amplified the complexity of PE management; advanced malignancy increased the risk of hemorrhagic complications, particularly given the patient’s anemia, potential for gastrointestinal bleeding, and prior anticoagulant therapy. Specific challenges in this case included balancing the dual risks of thrombosis and bleeding, especially when the patient required both mechanical thrombectomy and thrombolytic therapy, and addressing hypoxemic respiratory failure, a consequence of both PE and the underlying disease.

The "MOPETT trial" findings supported using reduced-dose alteplase as part of the therapeutic strategy. The reduced-dose protocol provided a middle ground, balancing the need for effective thrombolysis while minimizing bleeding risk. This approach was particularly relevant given the patient’s profile of severe hypoxemia and contraindications for standard thrombolysis when initial therapies failed.

Despite aggressive management of PE, the advanced stage of the patient’s malignancy and deteriorating performance status significantly reduced the likelihood of recovery from PE. They influenced decisions to prioritize palliative over curative goals. This underscores the value of early oncological evaluation, the careful weighing of risks and benefits in therapeutic decision-making, and the pivotal role of palliative care in providing support when curative options are no longer feasible.
